# The selection of chief residents across residency programs at a large academic medical center

**DOI:** 10.1186/s12909-023-04896-9

**Published:** 2023-12-08

**Authors:** Susan C. Mirabal, Scott M. Wright, Paul O’Rourke

**Affiliations:** 1 Medical Education Fellow in General Internal Medicine (GIM), Johns Hopkins University School of Medicine (SOM), Baltimore, MD USA; 2 Division of General Internal Medicine, 5200 Eastern Ave, MFL Center Tower Suite 2300, Baltimore, MD 21224 USA; 3 Johns Hopkins University SOM, and GIM Division Chief, Johns Hopkins Bayview Medical Center, Baltimore, MD USA; 4 Johns Hopkins University SOM, and Associate Program Director, Johns Hopkins Bayview Internal Medicine Residency Program, Baltimore, MD USA

**Keywords:** Graduate medical education, Chief resident, Selection practices

## Abstract

**Background:**

Chief residents have a unique role in graduate medical education (GME). They not only connect residents with program and hospital leadership, but also advocate for the wellbeing and educational priorities of trainees. Previous studies have focused on describing the characteristics of chief residents (CRs), however little is known about how CRs are selected across GME programs.

**Methods:**

One-on-one semi-structured interviews with all (*n* = 21) GME program directors at the Johns Hopkins University School of Medicine were conducted from January to March 2022. Investigators independently coded the transcripts using an inductive approach to categorize meaningful segments of text; this culminated in the identification of explanatory themes.

**Results:**

From discussions with 21 program directors, four themes were identified: (1) identifying candidates: timing, recruitment, nominations, as well as desirable attributes and data considered; (2) applications: expression of intent and participation in interviews; (3) selections: voting, discussions leading to consensus, and program director intimately involved in the choice(s); and (4) confidence in processes and outcomes.

**Conclusions:**

Our results provide a deeper understanding of the nuances associated with the selection of CRs. It is hoped that the descriptions of the similarities and differences across GME programs will prompt reflection about what is done at one institution such that all programs can consider what are the best practices to serve their individual goals and needs.

**Supplementary Information:**

The online version contains supplementary material available at 10.1186/s12909-023-04896-9.

## Background

Chief residents (CRs) are integral to graduate medical education (GME). They not only serve as exemplars within their training programs, but also play pivotal roles that shape the educational experience of trainees [1]. In the U.S.A., CRs have varied responsibilities depending on the training program specialty [2]. In addition to clinical responsibilities and administrative tasks such as scheduling clinical rotations, CRs may also be responsible for teaching, supervising, and evaluating residents and medical students [2, 3]. In one of their most vital capacities, CRs serve as a bridge linking the house staff to program and hospital leadership [1, 4]. Despite limited leadership experience in academic medicine, [5–7] chief residents are placed in a unique position to advocate for the wellbeing and working conditions of the house staff. Notable, a majority of chief residents in some specialties – including Pediatrics and Emergency Medicine - continue their commitment to the training of physicians by remaining engaged in academia and careers that keep them involved in education and scholarship [4, 8].

Given that chief residents are selected largely based on potential, previous studies have focused on identifying factors that may be predictive of success in the CR role [2, 9–11]. Characteristics such as strong communication and leadership skills coupled with unfailing professionalism are believed to be essential, [2] whereas performance on standardized exams (including U.S. Medical Licensing Exams or In-service Training Exams [ITE]) and having performed large numbers of procedures are not given as much consideration in selections for the chief resident role [10, 11]. While CRs also serve as representatives of the house staff cohort, recent studies reveal that often their demographic characteristics do not embody institutional commitments to diversity, equity, and inclusion (DEI) [12, 13].

Few publications have explored how chief residents are selected and the literature is devoid of best practices or guidelines to assist GME programs in selecting chief residents. Because specific institutions may have educational priorities and cultures that can be distinctive, we decided to perform a qualitative study to explore all aspects related to chief resident selection practices across GME programs at a single large academic institution. This approach is different from prior studies that explored CR selection practices among different residency programs within a single clinical specialty (e.g. Pediatrics or Dermatology), [11, 14] across several schools.

We hope that describing the approaches employed across programs at our school will generate insights and promote reflection about how best to handle the CR selection processes. Understanding the selection process may also help programs without defined CR roles to consider their value, and use thoughtful selection methods suited to address their programmatic goals if or when they elect to formalize such positions.

## Methods

### Aim

This qualitative study explored all facets related to CR selection practices across GME programs at a single large academic institution.

### Setting and participants

This study consisted of one-on-one interviews with program directors (PDs) of GME programs at the Johns Hopkins University School of Medicine in Baltimore, Maryland. All data collection occurred between January and March of 2022.

All GME programs with chief resident roles (*N* = 21) were identified by departmental websites. Program directors were e-mailed an invitation to participate in an audio-recorded semi-structured video interview. In the case that a program director was unavailable, an Associate PD (APD) was interviewed. Study informants provided verbal consent at the start of the interview.

### Study design

We conducted the interviews (Zoom video Communications, Inc., San Jose, California) using an interview guide (see Supplemental Content section) that was developed using an iterative process based on published literature and our own experience in GME. The interview guide was piloted with several program directors from different institutions who did not participate in the study.

Twenty-one interviews, lasting approximately 40 min, were conducted by one of the authors (S.M.). The interviewer (S.M.) ensured accuracy of all transcripts generated by Zoom and deidentified all transcripts before data analysis. Verbal informed consent was obtained from each participant after clarification of the study objectives and activities, and prior to interviews. This consent procedure was approved by the Johns Hopkins Medicine Institutional Review Board (IRB 00305985).

### Analysis

After using an ethnographic approach during data collection, the transcripts were analyzed using this methodology [15]. Deidentified transcribed interviews were uploaded to nVivo (QSR International, Burlington, MA) for data analysis. All transcripts were read by the authors to iteratively elaborate the coding template. All transcripts were then coded by two authors; the interviewer adjudicated noted differences in coding as needed. The team met to discuss meaningful concepts that led to the organization of the codes into themes and subthemes. During these meetings, the authors also selected representative quotes to be shared.

### Reflexivity

All authors are educators who have teaching roles in one GME program at our institution. P.O. serves as the APD for this same program. The PD of that program was interviewed for the study. The authors discussed their experiences and thoughts about CR selection; they considered their preferences and any potential biases. With coding being done iteratively, the authors considered all responses as being equally relevant and valid.

## Results

Representatives from all 21 GME programs with designated chief resident roles participated in the study. Select program characteristics are shown in Table 1.

**Table 1:** General characteristics of select GME training programs with designated chief residents (CRs) that participated in the study

Graduate Medical Education Programs	Type of program	Program size (approximate number of house staff per year, all PGY levels)	Years of post-graduate training (per specialty)	Extra year of training required prior to serving as CR	Typical number of CRs
Anesthesiology	Procedural	88	3	No	3
Dermatology	Procedural	22	3	No	2
Emergency Medicine	Procedural	47	4	No	3
Internal Medicine (2 programs at different hospitals): JH Hospital (JHH) and JH Bayview Medical Center (BMC)	Primary care	JHH: 150 JHBMC: 48	3	JHH CRs serve a year after completing residency training.	JHH: 4 JHBMC: 3
Neurology	Primary care	30	3	No	2
Obstetrics and Gynecology	Both	36	4	No	2
Ophthalmology	Procedural	20	4	Yes	1
Otolaryngology	Procedural	25	5-6.5	No	2
Pathology	Procedural	37	3–4	No	2
Pediatrics	Primary care	87	3	No	2
Physical Medicine and Rehabilitation	Procedural	27	3	No	3
Plastic Surgery (2 programs: Independent and Integrated)	Procedural	24	Independent: 3 Integrated: 7	No	Independent: 1 Integrated: 4
Psychiatry	Primary care	50	4	No	4
Radiology (2 programs: Diagnostic and Interventional)	Procedural	Diagnostic: 30 Interventional: 17	Diagnostic: 4 Interventional: 5	No	3
Radiation Oncology	Procedural	16	4	No	2

Analysis of coded transcripts resulted in the identification of four main themes: identifying candidates, applications, selections, and confidence in processes and outcomes. The total number of PDs or APDs commenting on themes and subthemes are shown in Table 2.

**Table 2:** Number of program directors commenting on themes and subthemes

Theme	Subthemes		Number of PDs commenting n,(%)
**Identifying candidates**	Processes	*Timing*	16 (76.2)
		*Recruitment*	17 (80.9)
		*Nomination*	16 (76.2)
	Attributes	*Clinical skills*	19 (90.5)
		*Teaching abilities*	19 (90.5)
		*Personal qualities*	21 (100)
		*DEI consideration*	14 (66.7)
	Data considered	*Objective criteria*	14 (66.7)
**Applications**	*Expression of intent*		8 (38.1)
	*Interviews*		6 (28.6)
**Selections**	*Voting*		15 (71.4)
	*Consensus through discussions*		11 (52.4)
	*PD intimately involved in the choice(s)*		11 (52.3)
**Confidence in processes and outcomes**			17 (80.9)

### Identifying candidates

In explaining the ways in which candidates are considered for the chief resident roles, our informants described three groups of ideas: the processes, the desirable attributes, and the data considered

### Processes

The specific ways to recognize potential candidates to serve as chief residents, including the timing, recruitment methods, and nomination strategies varied somewhat across the training programs.

#### Timing

Within some programs, the efforts to identify potential CRs begins in the first year of residency. Below is a representative quote:

*“Some of the interns see CR year as something that they want to participate in and they get involved right away, and then they stay involved the next couple of years, and they’re kind of obvious chief candidates.”* – PD # 8.

For others, efforts are concentrated in the year prior to assuming this role - as per the quote below:

*“We ask for the [chief resident application] submissions around January or February of their post-graduate year 2.”* – PD # 10.

#### Recruitment methods

Efforts to assess interest in the CR position were accomplished via multiple methods.

Many programs inquired as part of regular meetings with trainees, and others send e-mails to all or asked select candidates to apply. Below are representative quotes:

*“I emailed a bunch of people and said ‘Hey, I heard you might be interested…’ Because I wanted to make sure that they knew that they should run if they want, and they would have my support. And I did that to everyone who I had heard had asked any chief resident about being chief resident.”* – PD # 15.

*“There’s an email that goes out to all the eligible people, in their final year of training, asking anyone who’s interested to apply.”* – PD # 8.

#### Nomination strategies

Of the 21 PDs interviewed, 16 required that residents be nominated by anyone (or in some cases by specific individuals such as PDs, department chairs or faculty) in order to be considered for the CR role. The following quote represents this idea:

*“I send an email out to all residents and faculty. It reads: ‘It’s time to nominate people for chief resident, you can nominate yourself, or you can nominate someone else’.”* – PD # 9.

### Attributes

Program leadership asserted that strong clinical skills, solid teaching abilities, and specific personal qualities were highly desirable for the chief resident role.

#### Clinical skills

Most informants highlighted the importance of clinical performance in the consideration of future chief residents. This was assessed primarily by faculty evaluations, but ITE scores were also considered by a few programs to reflect clinical knowledge. Two representative quotes are shared below:

*“We’re looking for someone who [demonstrates] excellence in clinical competencies.”*– PD # 10

*“We want people who [are] doing well clinically and in their ITE, because we wouldn’t want to give them these additional responsibilities if their main focus should be on improving their knowledge base or clinical skills.”* – PD # 3.

#### ﻿Teaching abilities

Many were looking for chief residents who enjoyed and excelled at teaching; most also preferred those with academic career aspirations. Two illustrative quotes are shown here:

*“One of the main qualities we look for is, are they good at teaching…And if we get feedback … that [they] go out of the way to teach, then that’ll be really taken into consideration.”* – PD # 7.

*“I try to take that their [career] goals into account for the Chiefs. If it’s going to help them then and they’re excellent then we’ll ask them.”* – PD # 7.

Both clinical skills and teaching abilities were admittedly recognized to be largely subjectively assessed as described by the 2 quotes below:

*“We discuss among ourselves who’s done a good job on service, and who’s done a good job teaching – but those are subjective things.﻿”* – PD # 18.

*“To be honest, the subjective tends to trump all because we have very few objective measures… and it’s really their evaluations, which, of course, are themselves subjective.”* – PD # 19.

#### Personal qualities

Multiple personal qualities were highly valued in those being considered for CR. These included being a team player, displaying professionalism, respectfulness, and accountability. Leadership skills, including being organized and a good communicator were also brought up repeatedly. Representative quotes are shown here:

*“We’re looking for someone who is a good communicator, a good collaborator on a team, is able to articulate a vision and get people inspired to follow…We do look for excellence in the clinical competencies and the professionalism competencies.”* – PD # 10.

*“[CRs] often will get tasked with being the liaison between the general faculty and the residents, and so it has to be also somebody that’s really well respected by the faculty.”* – PD # 6.

*“It’s someone that has to be patient with the junior residents especially early on in the year. It has to be someone that’s kind and adaptable to changes.﻿”* – PD # 20.

*“We are looking for the ability to handle conflict, the ability to be organized, [and] the willingness to do the administrative work…we’re looking for people who are trusted and that often comes from some combination of temperament and intellect.﻿”* – PD # 16.

#### DEI consideration

Most program directors voiced a desire to prioritize DEI, however many explained that they were somewhat constrained by the relatively limited diversity in the resident class. Exemplary quotes follow:

*“In all that we do, [we] make sure that there’s diversity, equity and inclusion across all decisions.”* – PD # 19.

*“We don’t want the same type of people to do this job year after year. We want to make sure that we consider gender, race, ethnicity, etc. We have a small residency and we don’t have as many people to choose from as other residencies do.”* – PD # 20.

### Data considered

There was an acknowledgment that most of the data reviewed when selecting CRs was subjective – with only a paucity of objective criteria.

#### Objective criteria

Thoughts about objective versus subjective criteria are shown in a few representative quotes below:

*“I don’t think there are real objective measures, except for our faculty evaluations.”* – PD # 5.

*“I would say there’s no objective process[es] - it’s more like that he gets the gestalt of who’s a standout in the class and then asks them.”* – PD # 7.

### Applications

When it came to applying for CR, the processes were fairly consistent across programs and included expressing an intent to apply for the position with the willingness to participate in interviews.

#### Expression of intent

Residents were asked to submit materials, complete forms, and sometimes speak to their fellow residents about their interest in becoming CR. The informant below describes their own processes:

*“Any resident who wishes to run for chief should send us an email indicating that they would like to run, and a candidacy statement as to why they want the role.﻿”* – PD # 15.

#### Interviews

In a few programs, the interview was conducted by the APDs and/or PDs, while other programs involved a larger group to meet with candidates. Most often, the interviews were described to be standardized, however a few used unstructured formats that were more free-flowing. Below are two representative quotes:

*“We do round robin interviews. …The current chief residents, the program coordinators, and the program directors sit in 3 different rooms and the candidates rotate through each group.﻿”* – PD # 17.

*“We schedule six one-on-one interviews to hear about why they want the job, how they would handle personal stress, and their organizational skills. We have a series of questions we ask [everyone].﻿”* – PD # 15.

### Selections

Programs utilized a combination of methods to select finalists for CR. These include counting votes from residents and faculty members, achieving consensus through discussions by selection committees, and less frequently, the choice ultimately made by the program director. Each method was not mutually exclusive, for example, some programs utilized a combination of votes from the residents which was then reviewed by the PD and/or a selection committee prior to making a final decision.

#### Voting

Finalists were often determined by votes from residents and/or faculty rating as per the representative quote below:

*“We have a vote or election where we poll all faculty and residents and we add up the votes - whoever gets the most wins [chief resident].﻿”* – PD # 4.

Some PD/APDs felt that resident input was critical when choosing CRs:

*“The residents vote on [CRs]. We don’t give them any criteria that they have to consider. We tell them that they are going to represent you…The residents are mindful of the candidates’ strengths and weaknesses.﻿”* – PD # 1.

#### Consensus through discussions

Selection committees, composed of various members from program leadership, clinical evaluators, current CRs, faculty and others, engage in open and honest dialogue to pick candidate(s). In the quotes below, two program directors describe their selection committee and their process:

*“I sit down with my leadership team: my APDs, program manager, program coordinator and the current chiefs, and we discuss the candidates﻿”* – PD # 9.

*“[The PD] asks for input [from the] APD and the clinical competency committee, so it’s a group decision. A consensus.”* – PD # 20.

#### Program director intimately involved in the choice(s)

Program leadership, primarily the PD, sometimes makes the selection of CRs after considering the input from a selection committee and/or review of votes from the faculty and residents. The following quote illustrates this scheme:

*“The final say is the PD. [They] usually inform the chair and the vice chairs, but it’s more of an FYI, as opposed to [them] having veto power.”* – PD # 6.

### Confidence in processes and outcomes

Most informants reported substantial confidence in their processes and outcomes with respect to chief resident selection. Two representative quotes are shown here:

*“Short answer is, I like the way the process works.﻿”* – PD # 16.

*“We tried to make the process as fair and transparent as possible. We don’t advocate for any particular candidate. We let the faculty and the residents choose who they think will do the best job, and it’s worked out for us each time.﻿”* – PD # 21.

## Discussion

In this study, we describe the idiosyncrasies associated with the identification of candidates, the application processes, and the selection of chief residents across our institution’s GME programs. Considering that the majority of PDs were confident in their selection practices, we hope that our findings will encourage programs to reflect on their current methods and to identify best practices to serve their individual priorities and objectives.

Leadership role identity and emergence are important concepts in CR selection. Candidates with strong leadership role identities (i.e., they see themselves as leaders) may engage in relationship-building behaviors, placing them in central positions within their peer group [16]. This in turn makes them more likely to be viewed as leaders, [16] suggesting that the residents themselves may be particularly adept at identifying potential leaders among their peers. This was particularly evident in our findings, as some PDs emphasized the importance of resident input in selecting CRs. Integrating the residents’ preferences among the CR candidates gives credence to peer assessment and serves to democratize the process.

The methods used to select chief residents are not uniform across programs and the processes are not entirely transparent. Previous studies have described CR selection practices within individual GME programs, but not across multiple programs within a single institution [11, 12, 14, 17, 18]. These studies used surveys to gather PD responses and were limited by low response rates [10, 12, 14, 15]. The variable selection methods we observed are likely the result of individual programmatic goals and needs, perhaps compounded by the absence of standardized methods from governing bodies such as the Accreditation Council for Graduate Medical Education (ACGME). While the ACGME provides leadership and communication resources for newly appointed CRs, [19] it does not advise about best practices regarding CR selection processes. Without these guidelines, programs may unknowingly use biased methods with potentially significant implications that undermine the success of underrepresented minority physicians [20, 21]. Prioritizing inclusivity and limiting biases expands the candidate pool, promotes selection transparency, and may increase the chance of selecting the strongest candidate to fill these important educational leadership roles. Because some CRs remain involved academic medicine, [4, 8] placing greater emphasis on equity and diversity may help to retain and expand cohorts that have traditionally been underrepresented on the faculty [20, 21].

Consider the selection methods for Battalion Commanders in the U.S.A. Army, which long ago relied exclusively on a review of subjective performance evaluations and assignment history (or schedule) [22] — a rather similar process to CR selection with little to no objective criteria as described in our study. Under those processes, an officer could be appointed simply by having seniority and being ranked in the top 20% of their class [22]. No interest would be paid to candidates who possessed superior cognitive flexibility, cross-cultural fluency or interpersonal skills [22] – all vital characteristics of a successful oversea adviser and CR. The Army has since duly refined their approach by seeking candidates who possessed a combination of knowledge, skills, and behaviors; their reimagined selection criteria prioritizes strong communication skills, creativity, ethical leadership, and the ability to develop others [22]. Similarly, GME programs may benefit from re-defining the CR role to encourage the selection of exceptionally qualified individuals – as measured by more objective standards. Notably, the Army’s new criteria also increased the number of minority officers, at least in part due to bias-reducing strategies that guard against reflexive practices [22].

Several limitations of this study should be considered. First, this study was conducted at a single institution. Qualitative studies never generate generalizable findings. Our study of PDs across programs at one institution was a carefully chosen sampling decision given that different academic institutions can have unique educational values, cultures, and priorities. Second, descriptions of the CR selection process were obtained from PDs with varying levels of experience in their roles; for some their perspectives were restricted to only a few CR selection cycles but all were intimately involved with GME for lengthy periods of time. Finally, the study’s results serve to generate hypotheses about best practices for identifying candidates and selecting CRs. Quantitative studies that test various approaches would be needed to definitively identify the most effective practices.

## Conclusions

Our results reveal extensive details related to chief resident selection practices across GME programs. It is hoped that the descriptions of the similarities and differences across the programs studied will prompt reflection about current CR selection practices at a single institution and will encourage GME programs to consider best practices that may serve their programmatic goals.

### Supplementary Information

**Additional file 1.**

## Data Availability

The datasets generated and/or analyzed during the current study are not publicly available due to potential identification of participants, but are available from the corresponding author on reasonable request.

## Abbreviations

GME: Graduate medical education
CR: Chief resident
ITE: In–service training exam
DEI: Diversity, Equity, and Inclusion
PD: Program director
APD: Associate program director
ACGME: Accreditation Council for Graduate Medical Education
