# Supplementary material for: The selection of chief residents across residency programs at a large academic medical center
Source: BMC Med Educ. 2023 Dec 8;23:931. doi: 10.1186/s12909-023-04896-9 (PMC10709969; doi:10.1186/s12909-023-04896-9)
Supplement: Supplementary file 1 — Additional file 1. [file 12909_2023_4896_MOESM1_ESM.pdf]

SUPPLEMENTAL CONTENT

The Interview Guide

The interviewer introduces themselves and reads consenting script from IRB. There is a pause after soliciting questions. Consent is verified prior to proceeding.

Interviewer: We are interested in learning more about how chief residents are selected. We want to hear about your experiences and thoughts about this.

1. Please tell me the story and details leading up to the selection of your current chief residents.
2. Who is involved in the selection of chief residents?
3. What are some of the objective measures that are used and considered in the selection process?
4. What are some of the subjective elements that are used and considered in the selection process?
5. How are these objective and subjective criteria considered relative to one another?
    a. For example, if a candidate is objectively smarter or more clinically skilled and another is more personable and kinder, who is the better choice for your program – all else being equal.
6. How good is your process in choosing the right / best chief resident(s)?
7. How is diversity, equity and inclusion considered in chief resident selection?
8. What are other important aspects to understand regarding the chief resident selection process?
